# Mulberry and *Hippophae*‐based solid beverage attenuate hyperlipidemia and hepatic steatosis via adipose tissue–liver axis

**DOI:** 10.1002/fsn3.4155

**Published:** 2024-04-08

**Authors:** An‐Qi Zhu, Nin Luo, Ling‐Yue Sun, Xiao‐Ting Zhou, Shi‐Sheng Chen, Zebo Huang, Xin‐Liang Mao, Kun‐Ping Li

**Affiliations:** ^1^ Institute of Chinese Medicinal Sciences Guangdong Pharmaceutical University Guangzhou China; ^2^ Perfect Life & Health Institute Zhongshan Guangdong China; ^3^ Perfect (Guangdong) Co., Ltd. Zhongshan China; ^4^ School of Food Science and Engineering South China University of Technology Guangzhou China

**Keywords:** gene set enrichment analysis, hepatic steatosis, hyperlipidemia, liver–adipose axis, mulberry leaf

## Abstract

Dyslipidemia and hepatic steatosis are the characteristics of the initial stage of nonalcohol fatty liver disease (NAFLD), which can be reversed by lifestyle intervention, including dietary supplementation. However, such commercial dietary supplements with solid scientific evidence and in particular clear mechanistic elucidation are scarce. Here, the health benefits of MHP, a commercial mulberry and *Hippophae*‐based solid beverage, were evaluated in NAFLD rat model and the underlying molecular mechanisms were investigated. Histopathologic examination of liver and white adipose tissue found that MHP supplementation reduced hepatic lipid accumulation and adipocyte hypertrophy. Serum biochemical results confirmed that MHP effectively ameliorated dyslipidemia and decreased circulation‐free fatty acid level. RNA‐Seq‐based transcriptomic analysis showed that MHP‐regulated genes are involved in the inhibition of lipolysis of adipose tissue and thus may contribute to the reduction of hepatic ectopic lipid deposition. Furthermore, MHP upregulated ACSL1–CPT1a–CPT2 pathway, a canonical pathway that regulated mitochondrial fatty acid metabolism, and promoted liver and adipose tissue fatty acid β‐oxidation. These results suggest that adipose tissue–liver crosstalk may play a key role in maintaining glucose and lipid metabolic hemostasis. In addition, MHP can also ameliorate chronic inflammation through regulating the secretion of adipokines. Our study demonstrates that MHP is able to improve dyslipidemia and hepatic steatosis through crosstalk between adipose tissue and liver and also presents transcriptomic evidence to support the underlying mechanisms of action, providing solid evidence for its health claims.

## Graphical abstract

Animal tests demonstrate the efficacy of MHP, a mulberry and *Hippophae*‐based solid beverage, against high‐fat high‐fructose diet‐induced hyperlipidemia and hepatic steatosis. Transcriptomics reveals inhibition of lipolysis of adipose tissue pathway and upregulation of ACSL1–CPT1a–CPT2 pathway by MHP to promote fatty acid β‐oxidation. MHP also ameliorates metabolic inflammation by regulating the secretion of adipokines.

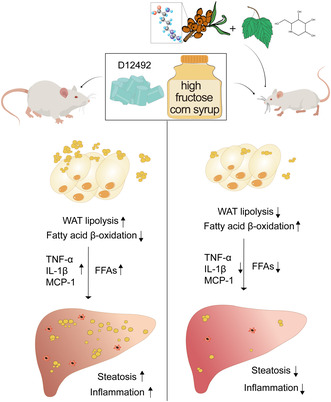

## 1 INTRODUCTION

Nonalcoholic fatty liver disease (NAFLD) is recognized as the primary cause of aberrant liver function as well as the most prevalent chronic liver disease worldwide, with a global incidence rate exceeding 25% (Younossi et al., 2020). NAFLD is characterized by the early‐stage accumulation of lipids (steatosis) in hepatocytes, which then can progress to nonalcoholic steatohepatitis (NASH) or more advanced liver pathologies such as fibrosis and cirrhosis (Powell et al., 2021). Numerous interventions have been employed in the efforts to combat NAFLD, while lifestyle modification, including dietary caloric restriction and exercise, remains the cornerstone of its therapy (He et al., 2023). However, owing to suboptimal adherence to this form of treatment, particularly with long‐term weight loss diets that might pose potential harm to the liver, there is a notable focus on identifying novel therapeutic agents for the management or prevention of NAFLD progression (Rong et al., 2022). Although breakthroughs have been made on the pathogenetic mechanisms of NAFLD, until now FDA‐approved drugs for NAFLD (NASH) are rare (Paternostro & Trauner, 2022). Some chemically synthesized drugs for body weight control as well as for blood sugar and lipid management are found to bring healthy benefits for NAFLD patients but have no satisfied results (Attridge et al., 2013; Pinal‐Fernandez et al., 2018). Interestingly, extensive investigations on bioactive dietary substances against NAFLD in the past decade suggest that phytonutrients have great potential to improve treatment efficiency of NAFLD. Therefore, the intake of food‐sourced natural products is a promising strategy in addressing the worldwide health challenges related to NAFLD (Hossein Zadeh et al., 2023; Tian et al., 2021). However, there is still a deficiency in understanding the mechanisms of action of these products (Bagherniya et al., 2018; Jang et al., 2018). Further studies on the underlying molecular mechanisms of potential supplements against the development of NAFLD are needed to provide better support for their precise use (Xu et al., 2020).

In our pilot test, some commercially available dietary extracts have shown lipid‐lowering activities. Among them, the solid beverage MHP, which mainly consists of mulberry leaf aqueous extract and *Hippophae* protein peptides, showed protective potential against obesity and hyperlipidemia induced by a high‐fat diet in cellular and animal models. Interestingly, mulberry leaf has been traditionally used as a herb for lowering blood glucose and lipids, consistent with its high content of flavonoids and alkaloids (Liu et al., 2023; Zhang et al., 2022). On the other hand, *Hippophae* protein peptides are functional ingredients from *Hippophae rhamnoides* L. seeds, which have been shown to enhance insulin sensitivity and reduce lipogenesis (Wang et al., 2022; Yuan et al., 2016). However, their protective potential against NAFLD and underlying mechanisms have yet to be fully elucidated.

Transcriptome analysis is emerging as a robust tool in the investigation of fundamental pathogenic mechanisms and discovery of therapeutic targets. It offers insights beyond phenotypic observations and provides valuable clues for further in‐depth studies (Perakakis et al., 2020; Zhang et al., 2023). In this study, we first confirmed the improvement effects of MHP against dyslipidemia and hepatic steatosis using a high‐fat high‐fructose (HFF) diet‐induced rat model. Then, transcriptomic analysis of liver and adipose tissues was performed to investigate the hepatic and adipose gene expression patterns in the rat models with or without MHP supplementation. Based on this, the potential molecular mechanisms were analyzed by gene set enrichment analysis (GSEA). Our results revealed that MHP inhibited the lipolysis of adipose tissue, upregulated ACSL1–CPT1a–CPT2 pathway, and promoted fatty acid β‐oxidation. MHP was also shown to ameliorate metabolic inflammation through regulating the secretion of adipokines.

## 2 MATERIALS AND METHODS

### 2.1 Preparation and characterization of MHP

MHP was a mulberry and *Hippophae*‐based solid beverage. Its main components were mulberry leaf aqueous extract, *Hippophae* protein peptide, and L‐arabinose. MHP was produced in a pilot plant that adhered to the strict guidelines of Good Manufacturing Practice, which follows specific quality assurance protocols for nutritional ingredients, active compounds, heavy metals, and microorganisms (See Tables S1 and S2 and Figure S1 in Supplementary Materials).

### 2.2 Animal experiment

All animal experiments were approved by the Institutional Animal Care and Use Committee of the Guangdong Pharmaceutical University (Guangzhou, China), with the approval number SPF2022011. All animal studies were conducted in accordance with the ARRIVE (Animal research: reporting of in vivo experiments) guidelines (Kilkenny et al., 2010). Male SD rats (5 to 6 weeks old) were supplied by Guangdong Medical Laboratory Animal Center (Guangzhou, China) and housed in a specific pathogen‐free (SPF) facility with 12‐h day and night cycles at 22–24°C and 60%–65% humidity. After 1 week of acclimation on a normal chow diet, the rats were randomly divided into two groups. One group was fed a normal diet (Cont group, *n* = 8) and another group was fed a high‐fat high‐fructose diet (Li et al., 2022), and 2 weeks later the abnormal weight rats were excluded. Then, the high‐fat high‐fructose group was divided into HFF group, MHP‐L group, and MHP‐H group (*n* = 8 for each group). The normal chow provided 23% of calories from protein, 65% of calories from carbohydrate, and 12% of calories from fat, and a digestible energy of 3.40 Kcal/g. The high‐fat diet provided 20% of calories from protein, 20% of calories from carbohydrate, and 60% of calories from fat, and a digestible energy of 5.24 Kcal/g (D12492, Research Diets Inc., New Brunswick, NJ, USA). The HFCS‐sweetened drink, a 12.5% fructose solution diluted from HFCS‐55 with water, was supplied in a separate bottle (3.54 Kcal/mL). In the following 8 weeks, each rat enjoyed its diet as before, whereas MHP‐L group and MHP‐H group were supplemented with MHP at a dose of 875 mg/kg/day and 5250 mg/kg/day, respectively. Body weight was assessed twice per week. At week 10, all rats were anesthetized with pentobarbital sodium and sacrificed. Blood, liver, epididymis white adipose tissue (eWAT), and inguinal white adipose tissue (iWAT) were collected. Part of the tissues was fixed with 4% paraformaldehyde (PFA) for histological examination, while the remaining tissues were snap‐frozen in liquid nitrogen and stored at −80°C for subsequent RNA‐Seq.

### 2.3 Histopathologic evaluation

Hematoxylin and eosin (H&E) staining of liver tissue, eWAT, and iWAT were carried out as described previously (Li et al., 2022). Briefly, rat liver tissues and adipose tissues were washed with cold saline solution and fixed in 4% paraformaldehyde followed by paraffin embedding. Then, the samples were cut into 5‐μm‐thick sections and stained with H&E. The frozen sections of liver tissue were stained by oil red O (ORO) to show lipid droplets. Pathological changes in the liver and adipose tissues were evaluated by a light microscope of PerkinElmer Vectra 3 (PerkinElmer, USA). The areas of liver lipid droplets were analyzed using Image Pro Plus 6.0 software (Media Cybernetics, USA).

### 2.4 Biochemical analysis

The levels of serum triglyceride (TG), total cholesterol (TC), high‐density lipoprotein cholesterol (HDL‐C), and low‐density lipoprotein cholesterol (LDL‐C) were determined with commercial kits (Nanjing Jiancheng, China) based on the manufacturer's instructions. Serum monocyte chemoattractant protein‐1 (MCP‐1), tumor necrosis factor‐alpha (TNF‐α), and interleukin‐1 beta (IL‐1β) were measured using the corresponding commercial enzyme‐linked immunosorbent assay (ELISA) kits (CUSABIO, Wuhan, China).

### 2.5 Free fatty acid analysis

As we previously described (Li et al., 2018), quantitative profiling of free fatty acids (FFAs) was performed on 7890B‐5977B GC–MS Systems (Agilent Technologies, USA) with an Agilent DB‐23 column (60 m × 0.25 mm × 0.15 μm, Agilent, MA, USA). Briefly, for all samples, CHCl_3_‐MeOH (2:1, v/v) solution was used as extraction solvent and the lower organic layer was collected as the total lipid fraction. FFAs were fractionated by HPTLC silica gel plates from the total lipid fraction as described (Burdge et al., 2000) with modification. Silica strips containing FFAs were scraped and dispersed in 100 μL of CHCl3: methanol (2:1, v/v), vortexed for 30 s, and centrifuged at 1000 *g* for 2 min at room temperature. Then, the supernatant was collected and derivatized prior to GC–MS analysis (see Figure S2 in the Supplementary Materials for detailed information).

### 2.6 Transcriptomics analysis

Transcriptomics analysis was performed as described previously (Li et al., 2022). Briefly, total RNA from the liver and adipose tissue was extracted using commercial Trizol reagent kits (Promega, USA) following the manufacturer's protocol. The cDNA libraries were constructed and sequenced on Illumina Novaseq6000 by Gene Denovo Co. (Guangzhou, China). All the RNA‐Seq reads were furtherly filtered and standard transcriptomics analysis was carried out using R bioconductor and DESeq2 package. Genes that passed a threshold of *p* values <.05 and |fold change| > 1.5 were regarded as differentially expressed genes (DEGs) and considered for further analysis. Gene set enrichment analysis (GSEA) was implemented on the Java GSEA platform. For each KEGG biological pathway, the genes involved in were defined as a gene set, and then a ranked list and a ‘gene set’ permutation type of the gene set were generated. Gene sets with NES absolute values >1, *p* values <.05, and FDR values <.25 were considered statistically significant.

### 2.7 Western blotting

The liver and adipose tissue underwent lysis by utilizing RIPA buffer (NCM Biotech) under ice‐cold conditions, and then homogenized and centrifuged at 4°C and 15200 *g* for 10 min. Successively, the total protein samples were carefully prepared from the supernatant. The protein concentration was conducted utilizing a BCA protein assay kit (Beyotime, Beijing, China). Subsequently, the proteins were separated by sodium dodecyl sulfate–polyacrylamide gel electrophoresis (SDS‐PAGE) and transferred onto a PVDF membrane. After blocking with 5% skim milk in TBST for 2 h, the membrane was subjected to incubation with primary antibodies against ACSL1 (ab177958), CPT1A (ab128568), and CPT2 (ab181114) supplied by Abcam company (Cambridge, UK) and β‐actin (Cell Signaling Technology, Danvers, USA) at 4°C overnight, respectively, and the membrane was incubated with appropriate HRP‐conjugated secondary antibody at room temperature for 1 h. Visualization was carried out using a BeyoECL Plus kit (Beyotime, China). Protein band detection was performed on Universal Hood II Chemiluminescence Imaging System (Bio‐Rad, Hercules, CA, USA).

### 2.8 Statistical analysis

All data are expressed as means ± standard errors of the means (SEM). A one‐way analysis of variance (ANOVA) was performed. *p* < .05 was noted as significant. R ggplot2 and R pheatmap package (https://www.rproject.org) and GraphPad Prism 6.0 software (GraphPad, California, USA) were used for graphics.

## 3 RESULTS

### 3.1 MHP ameliorates dyslipidemia and hepatic steatosis in NAFLD rat model

**FIGURE 1:**
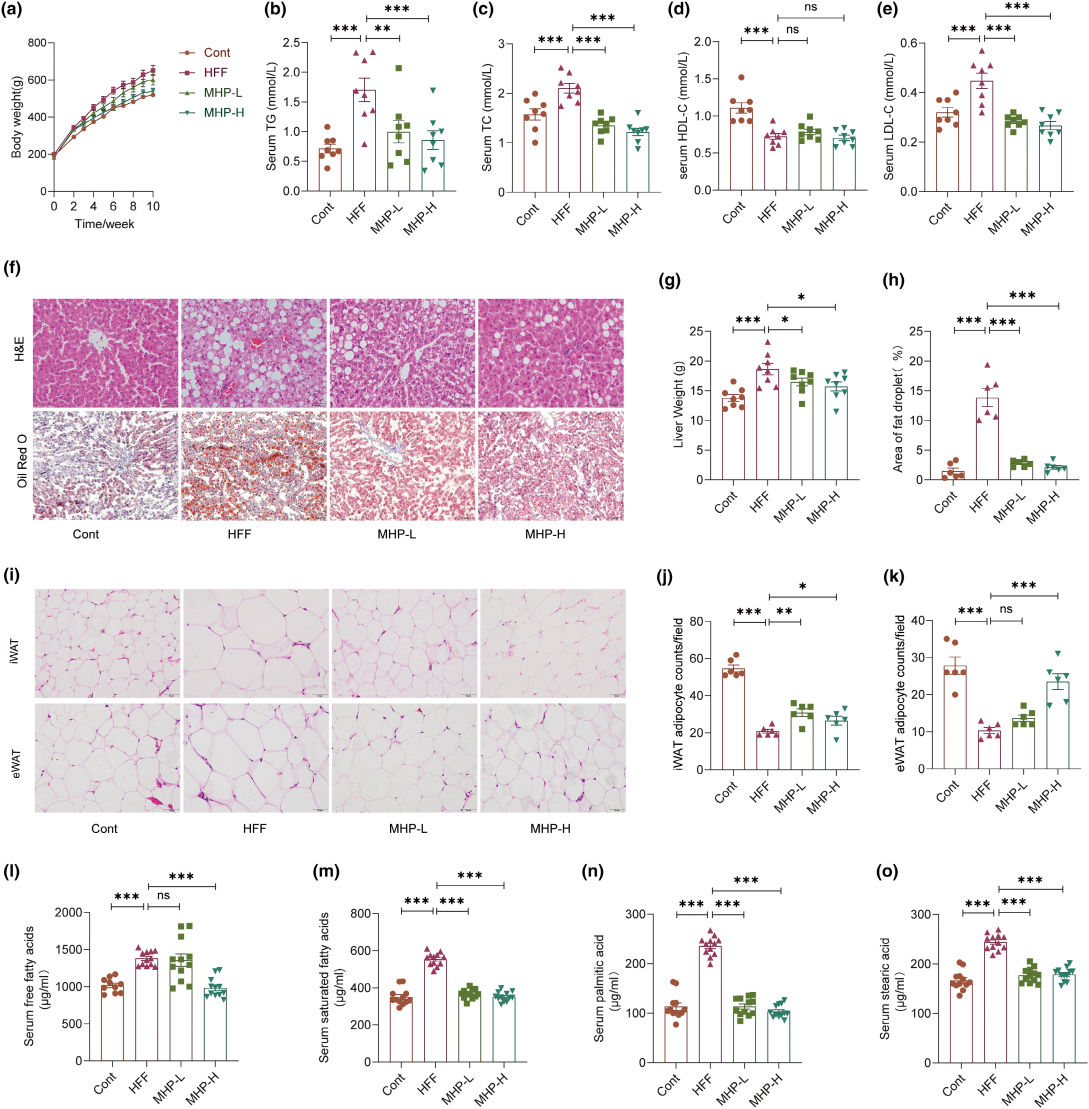
MHP ameliorates HFF‐induced dyslipidemia and hepatic steatosis in SD rats. (a) Body weight change curve; (b–e) Level of serum TG, TC, HDL‐C, and LDL‐C; (f) H&E staining (200×) and oil red O staining (100×) of liver tissue sections; (g) Liver weight; (h) Quantification of lipid drop area; (i) H&E staining of iWAT and eWAT sections; (j, k) iWAT and eWAT adipocyte counts per field of sections observed by light microscope; (l,m) Serum total FFAs and saturated FFAs; (n, o) Serum palmitic acid and stearic acid level. Data are expressed as mean ± SEM (*n* = 6–8), the serum‐free fatty acid analysis was repeated twice. *p < .05, **p < .01, ***p < .001 versus HFF group. MHP, mulberry and *Hippophae*‐based solid beverage.

Given that most NAFLD patients are overweight or obese, this study employed the high‐fat high‐fructose diet‐induced NAFLD rat model, as previously described (Li et al., 2022), to examine the efficacy of dietary MHP. As shown in Figure 1a, HFF‐induced body weight increase was suppressed by MHP administration, while MHP‐H reduced the body weight gain to a greater extent than MHP‐L. As overconsumption of HFF diet can lead to a dysregulation of carbohydrate and lipid metabolism (Li et al., 2022), we assessed the serum parameters related to lipid metabolism. As shown in Figure 1b–e, the serum TG, TC, and LDL‐C levels were significantly decreased after 8 weeks of MHP supplementation, while serum HDL‐C levels had no significant difference as compared with HFF‐fed rats, indicating that the high dose of MHP supplementation resulted in the alleviation of abnormal serum lipid metabolism.

It is known that excess lipid in circulation results in lipotoxicity and plays a role in the progression of NAFLD. As accumulation of excess lipids within hepatocytes is a well‐recognized hallmark of NAFLD, we performed pathological evaluation of the liver tissue. H&E and oil red O staining revealed an increased presence of lipid droplets in the liver tissues of HFF group rats, whereas MHP supplementation reduced the hepatic lipid accumulation and its high dose was more effective and the same result was observed, with MHP also significantly reducing liver weight (Figure 1f–h). Based on the H&E staining results of white adipose tissues, it can be observed that HFF diet intake led to more hypertrophic adipocytes, while MHP intervention alleviated the pathological changes (Figure 1i–k). Consistent with this result, MHP also significantly reduced fat mass (Figure S3). It has been suggested that accumulation of saturated FFAs in hepatocytes correlated with NAFLD progression (Kartsoli et al., 2020). Herein, a GC–MS‐based lipidomic profiling of serum was carried out and MHP‐H was confirmed to decrease the level of serum total FFAs and saturated FFAs, in particular palmitic acid and stearic acid content (Figure 1l–o).

### 3.2 MHP reprograms liver transcriptional landscape of NAFLD rats

**FIGURE 2:**
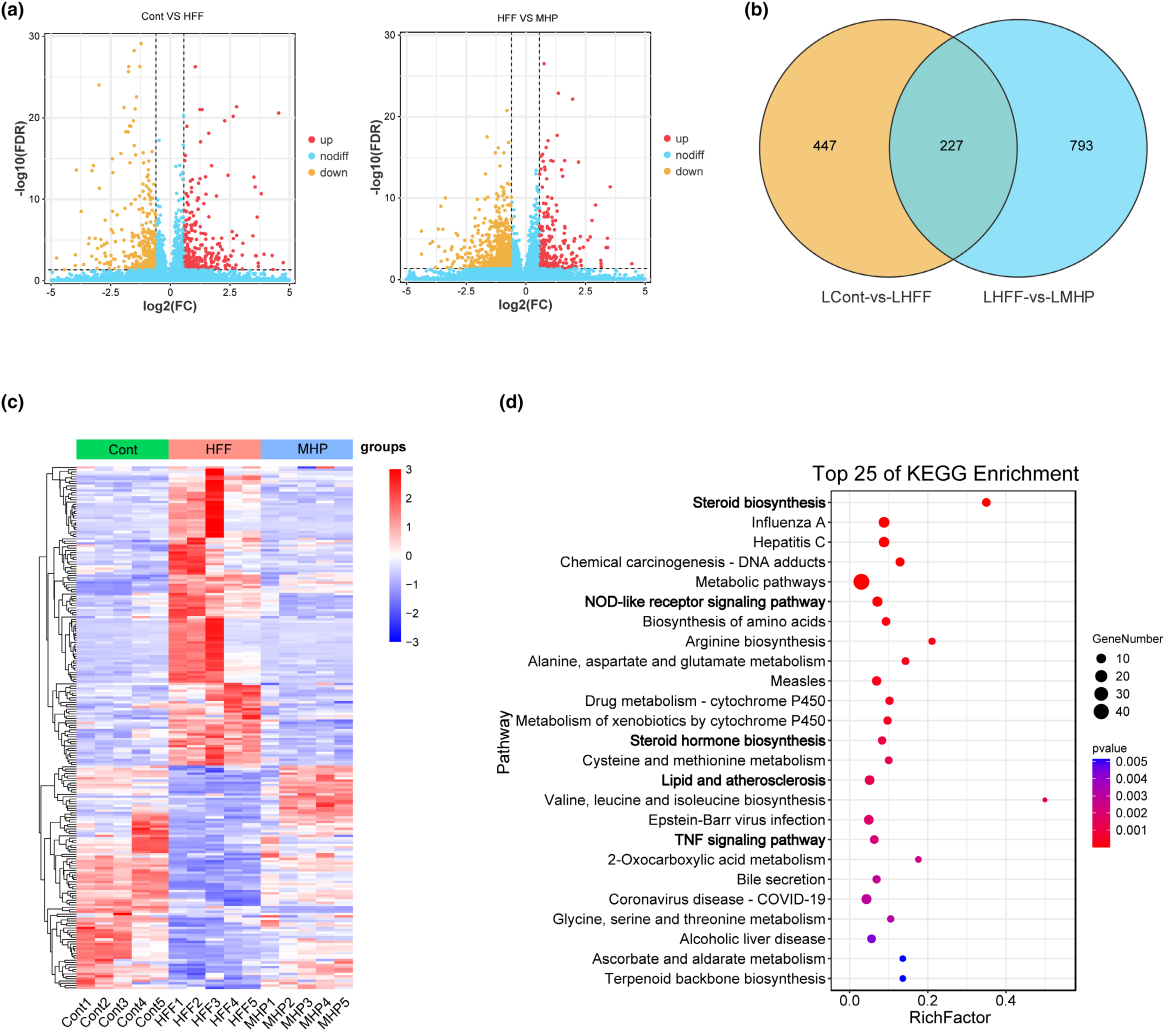
MHP reverses HFF‐induced hepatic transcriptome alterations. (a) Volcano plot analysis of significantly different genes in Cont versus HFF and HFF versus MHP; (b) Venn diagram of the common DEGs in the two cases of Cont versus HFF and HFF versus MHP; (c) Heatmap of DEGs among the three different groups; (d) Top 25 pathways according to KEGG enrichment pathway analysis of the common DEGs. *n* = 5 per group.

As shown above, better efficacy was observed in the high‐dose treatment of MHP, and thus MHP‐H was chosen for further investigations. As a growing number of studies indicate that transcriptomics has practical applications in phytomedicine development (Koks et al., 2021), we performed transcriptomic analysis to investigate the mechanism of MHP. To investigate the impact of MHP on the global gene expression of rats, hepatic transcriptomics analysis was performed. As shown in Figure 2a, there existed an obvious alteration among the hepatic transcriptome of the three groups of rats. Applying a threshold of fold change >1.5 and *p*‐value <.05, a total of 1467 DEGs were screened out. In brief, there were 674 DEGs between the control and HFF group, 1020 DEGs between the HFF and MHP group, and 227 DEGs were shared among the three groups (Figure 2b). Hierarchical clustering analysis was subsequently conducted based on the expression levels to delineate distinctions in the expression patterns of DEGs among the three groups (Figure 2c). The heatmap of DEGs revealed an altered gene expression pattern between the control group and HFF group, while MHP could reverse HFF‐induced hepatic transcriptome alterations. To investigate the potential roles of the DEGs, we conducted functional enrichment analysis, revealing a significant enrichment of DEGs in lipid metabolic pathways, for example, steroid biosynthesis, steroid hormone biosynthesis, lipid and atherosclerosis, and inflammatory signaling pathways such as NOD‐like receptor signaling pathway and TNF signaling pathway (Figure 2d). These results suggest that the efficacy of MHP is associated with its regulation of lipid metabolism and inflammatory pathways.

### 3.3 MHP reduces hepatic lipid accumulation through inhibiting lipolysis of adipose tissue

**FIGURE 3:**
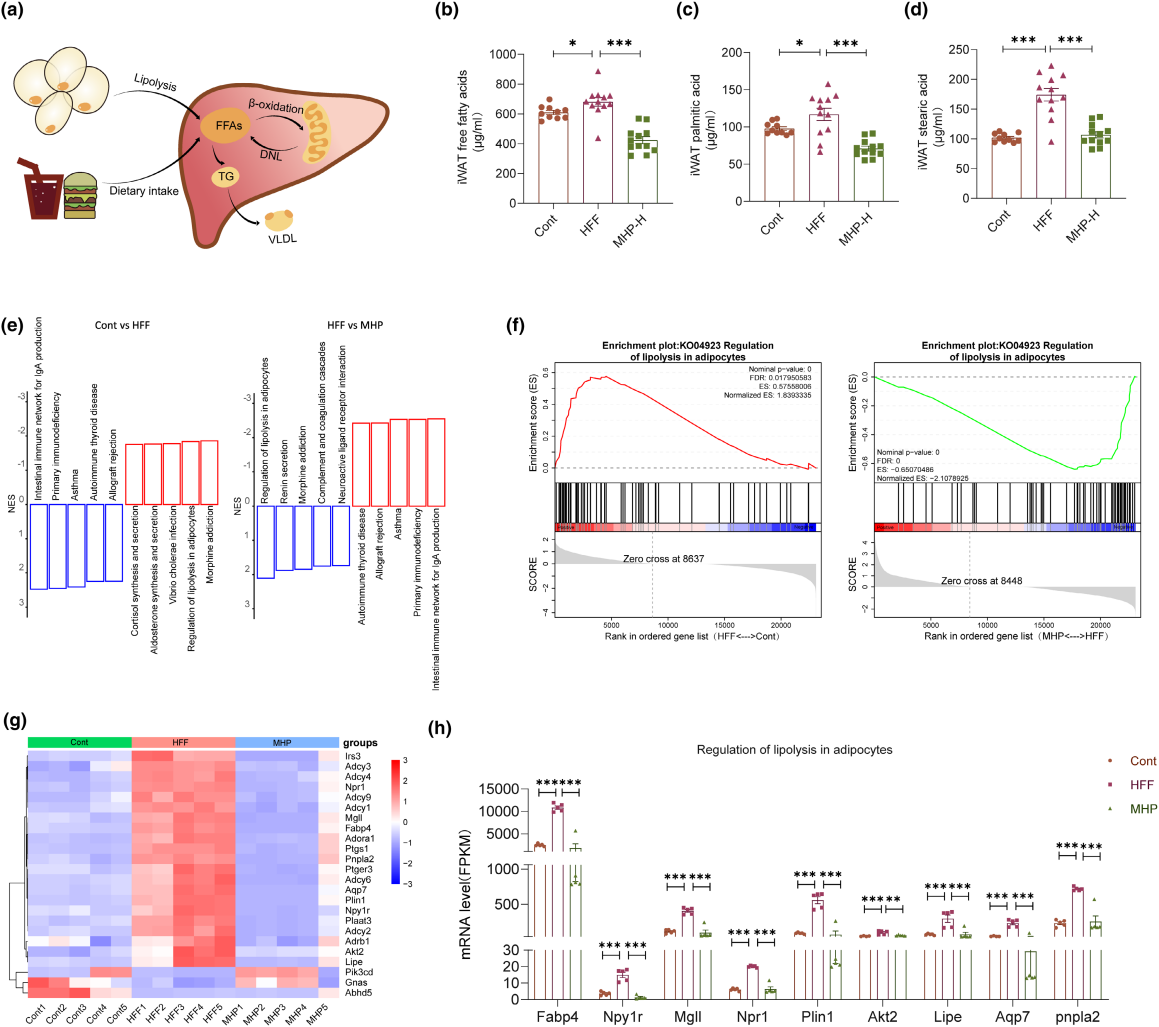
MHP ameliorates hepatic steatosis through inhibiting lipolysis of adipose tissue. (a) Major cellular processes that contribute to the hepatic pool of FFAs in NAFLD; (b–d) Total FFAs, palmitic acid, and stearic acid levels in iWAT; (e) Top 10 significant gene sets sorted on normalized enrichment scores (NES) for Cont versus HFF and HFF versus MHP; (f) GSEA enrichment plot: KO04923 regulation of lipolysis in adipose; (g) Heatmap of the expressions of lipolysis pathway‐related genes; (h) mRNA level of lipolysis pathway‐related genes. Gene expression data are expressed as mean ± SEM (*n* = 5). Free fatty acid analysis of iWAT was repeated twice (*n* = 7–8). *p < .05, **p < .01, ***p < .001 versus HFF group.

Under normal conditions, apart from dietary fat and hepatic de novo lipogenesis (DNL), fatty acids released from adipose tissue constitute the primary origin of hepatic lipids (Duwaerts et al., 2017). However, impaired fat storage capacity due to dysfunctional adipose tissues can lead to the accumulation of ectopic fat in the liver and other organs. This condition triggers oxidative stress in hepatic mitochondria, thereby hastening the progression of NAFLD (An et al., 2021). A contributing factor to the accumulation of lipids in the liver is the excessive release of nonesterified fatty acids (NEFAs) resulting from lipolysis in dysfunctional adipose tissue (Figure 3a). Therefore, FFA profiling of iWAT was performed. It was observed that MHP can ameliorate the total FFAs, palmitic acid, and stearic acid contents in iWAT (Figure 3b–d). Then, we performed GSEA analysis on the adipose tissue transcriptome (Figure 3e). The top five upregulated and downregulated KEGG pathways were selected for further analysis, and the adipose tissue lipolysis pathway was found significantly upregulated in the HFF group. After treatment with MHP, the pathway was significantly downregulated as compared to HFF group, which was consistent with the results of free fatty acid analysis (Figure 3f). The heatmap of DEGs related to the lipolysis pathway also showed that MHP improved the dysregulation of lipolysis gene expression induced by HFF diet (Figure 3g). As shown in Figure 3h, the key genes involved in the adipose tissue lipolysis pathway, including *Fabp4*, *Mgll*, *Plin1*, *Lipa*, and *Pnpla2*, had a significant upregulation trend in the HFF group as compared with the control group, while MHP supplementation reversed the tendency. Together, these results indicate that the HFF diet induces lipolysis of adipose tissue as compared with the control, and supplementation of MHP reduces lipid accumulation in liver through inhibiting lipolysis of adipose tissue.

### 3.4 MHP improves hyperlipidemia and hepatosteatosis by regulating fatty acid β‐oxidation pathway

**FIGURE 4:**
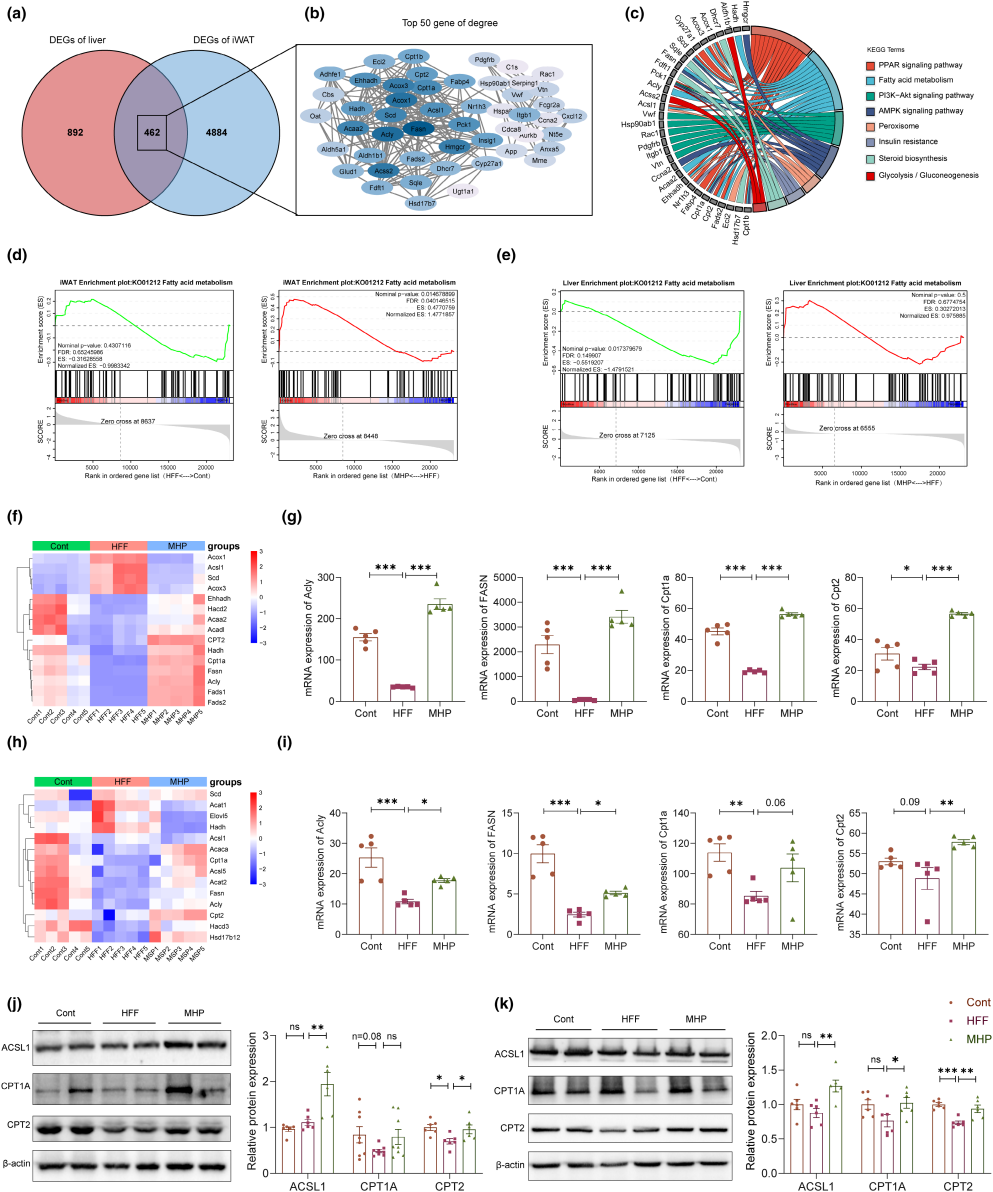
MHP promotes fatty acid β‐oxidation in white adipose tissue and liver. (a) Venn diagram of the common DEGs between the liver and iWAT; (b) The STRING protein interaction network generated from the top 50 DEGs; (c) Enrichment chord plot of the top 50 genes sorted in STRING; (d) GSEA analysis of fatty acid metabolism pathway of iWAT; (e) GSEA analysis of fatty acid metabolism pathway of liver; (f) Heatmap of the expressions of fatty acid metabolism pathway‐related genes of iWAT; (g) Expression level of fatty acid metabolism pathway relative genes of iWAT (*n* = 5); (h) Heatmap of the expressions of fatty acid metabolism pathway‐related genes of liver; (i) Expression level of fatty acid metabolism pathway relative genes of liver (*n* = 5); Western blotting of ACSL1, CPT2 (*n* = 6), and CPT1A (*n* = 8) in iWAT (j) and liver tissue (*n* = 6) (k). Data are expressed as mean ± SEM. *p < .05, **p < .01, ***p < .001 versus HFF group.

Almost all effective treatments for hyperlipidemia and hepatic steatosis are known to involve reduction of adiposity, indicating that the liver–adipose tissue metabolic axis is essential to NAFLD prevention (Stephenson et al., 2022). In the present study, an integrated analysis of liver and iWAT transcriptomes was performed to investigate the responses of liver–adipose axis to HFF challenge and MHP intervention. As shown in Figure 4a, there were 892 DEGs and 4884 DEGs for liver and iWAT, respectively, and 462 common DEGs in both transcriptomes. When the common DEGs were uploaded for STRING protein interaction network analysis, *Fasn*, a key gene for DNL, was found to be located in the center of the network (Figure 4b). Furthermore, through gene enrichment chord diagram analysis, we observed that the top 50 DEGs sorted by STRING were predominantly enriched in PPAR, fatty acid metabolism, and PI3K‐AKT signaling pathways in the KEGG database (Figure 4c).

Except for the intake of FFAs from the circulation, DNL and fatty acid β‐oxidation are also important factors affecting the fatty acid pool (Donnelly et al., 2005). Thus, GSEA analysis of the liver and iWAT transcriptomes was performed to analyze fatty acid metabolism in different tissues. As compared with the control group, the iWAT fatty acid metabolic pathway showed a downward trend in HFF group but a normal tendency in MHP‐treated group (Figure 4d). Similar results also appeared in liver tissue (Figure 4e). The heatmap of genes associated with fatty acid metabolism revealed a conspicuous distinction between the control and HFF groups but appeared to be normal in the MHP group (Figure 4f,h). For instance, expressions of the DNL‐related genes *Acly* and *Fasn* and the fatty acid β‐oxidation‐related genes *Cpt1a* and *Cpt2* were significantly downregulated in the HFF group as compared to the control, where these genes were all significantly upregulated in the MHP‐treated group as compared to the HFF group (Figure 4g,i). Furthermore, the protein levels of ACSL1, CPT1A, and CPT2 were measured by western blotting technique (Figure 4j,k). Interestingly, macronutrient metabolism is known to be a highly coordinated process, in which adipose tissue–liver axis executes a central role in nutrient absorption, processing, transportation, and storage (Duwaerts & Maher, 2019). Therefore, improvement of hepatic lipid accumulation and adipocyte enlargement by MHP is likely due to the promotion of fatty acid oxidation, eliminated metabolic adaptation, and restored DNL level in rats.

### 3.5 MHP ameliorates inflammation in liver through regulating the secretion of adipokines

**FIGURE 5:**
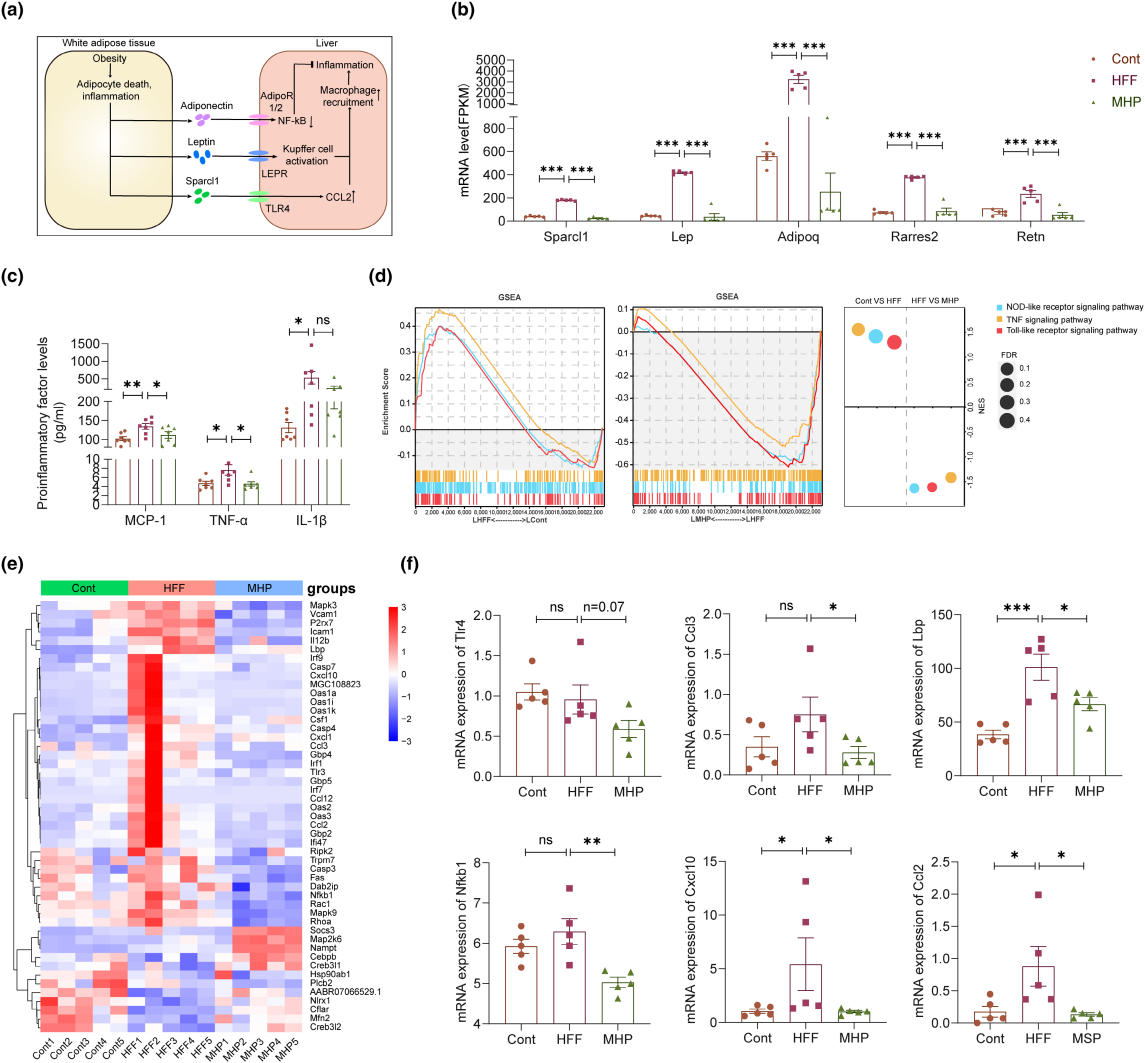
MHP ameliorates inflammation in liver by regulating the secretion of adipokines. (a) Schematic liver–adipose axis alterations under the pathological condition of NAFLD; (b) mRNA expression level of adipokines; (c) Serum level of MCP‐1, TNF‐α, and IL‐1β (*n* = 7); (d) GSEA analysis of hepatic inflammatory signaling pathway; (e) Heatmap of the expressions of inflammatory signaling pathway‐related genes in liver; (f) mRNA expression level of inflammatory signaling pathway‐related genes in liver. Data are expressed as mean ± SEM (*n* = 5). *p < .05, **p < .01, ***p < .001 versus HFF group.

It has been established that the liver–adipose axis plays a critical role in maintaining the whole body's metabolic homeostasis (Duwaerts & Maher, 2019). As shown in Figure 5a, WAT contributes to the development of all aspects of NAFLD by releasing fatty acids, proinflammatory cytokines, and various polypeptides. As diseased adipocytes also undergo modifications in the production of adipokines, the mRNA levels of leptin, adiponectin, and sparcl1 were measured (Figure 5b). In the HFF group, the mRNA expression levels of adipokines, including *Sparcl1*, *Lep*, and *Adipoq*, were significantly elevated. After treatment with MHP, the secretion of adipokines returned to normal levels. Likewise, the upregulated serum level of the inflammatory factor MCP‐1, TNF‐α, and IL‐1β in the HFF group was decreased to a normal level as the control after MHP supplementation (Figure 5c). Then, we performed GSEA analysis on the liver tissue transcriptome and found that the inflammatory pathways, including TNF signaling pathway, NOD‐like receptor signaling pathway, and Toll‐like receptor signaling pathway, were upregulated after HFF diet feeding but downregulated after MHP treatment (Figure 5d). The heatmap of DEGs related to the inflammatory pathways also suggested that MHP improved the dysregulation of inflammatory gene expression induced by HFF diet (Figure 5e). As shown as Figure 5f, the key genes involved in inflammatory pathways in the model group, including *Ccl2, Lbp, Nfkb1*, *Ccl3*, and *Cxcl10*, showed a significant upregulation trend as compared with the control group and a significant downregulation trend after MHP treatment. Moreover, we observed a significant downward in the expression of *Tlr4* following MHP administration. Collectively, these results indicate that MHP could attenuate inflammation in liver tissue through regulating the secretion of adipokines.

## 4 DISCUSSION

Although NAFLD/NASH is now a global health issue, the pathogenesis that results in lipid accumulation, cell apoptosis, inflammation, and fibrosis is yet to be comprehensively elucidated, while pharmacological treatments—completely safe and effective—are not currently known. Nevertheless, an increasing number of studies have been done on bioactive phytochemical compounds, for example, hesperidin (Morshedzadeh et al., 2023), naringenin (Cao et al., 2023), and curcumin (Baziar & Parohan, 2020), for their potentials to prevent the onset and progression of NAFLD (Frasinariu et al., 2022). As a result, plant‐based dietary supplements are growingly recognized to play an important role in the management of NAFLD, especially at its initial stage (Perumpail et al., 2018). For example, sea buckthorn is found to reverse obesity induced by a high‐fat diet and promote fat browning through the activation of AMPK/SIRT1 pathway (Wang et al., 2022). Interestingly, mulberry leaf has been previously shown to have hepatoprotective effect likely through regulating adipocytokines, inflammation, and oxidative stress in a high‐fat diet‐induced mice model of obesity and NAFLD (Ann et al., 2015; Peng et al., 2018). In this study, MHP, a mulberry and *Hippophae*‐based solid beverage, was confirmed to ameliorate hyperlipidemia and ectopic lipid deposition at a systemic level in rat model of NAFLD.

In fact, a prolonged consumption of HFF diet can result in obesity (Malik & Hu, 2022). Generally, obesity is associated with a disturbed adipose tissue function characterized by adipocyte hypertrophy, an impaired lipolysis, and pro‐inflammatory phenotype, which contributes to insulin resistance (IR) (Rohm et al., 2022). Adipocytes are among the most insulin‐sensitive cells. When adipocytes become dysfunctional, they are resistant to the antilipolytic effect of insulin, leading to more FFAs being released into the bloodstream that play a key role in the development and maintenance of IR (Rosso et al., 2019). In this study, a 10‐week HFF diet intake was found to induce IR in rats, which was improved by MHP intervention (Figure S4). This was consistent with changes in serum FFA levels.

In general, adipose tissue is appreciated for its lipid storage role, but overflowed FFA will redirect toward other organs, most notably the liver, once its intrinsic limit is reached (Navab et al., 2008). In this case, hepatocytes store the extra lipids, primarily in the form of triglycerides, resulting in hepatic steatosis. To be more specific, an excess presence of circulating FFAs, arising from increased lipolysis and decreased uptake of fatty acids in subcutaneous adipose tissue, has the potential to result in the accumulation of ectopic fat, such as in the liver and skeletal muscle. The consensus is that lipotoxicity induced by FFAs significantly contributes to the pathogenesis of NAFLD, and mitigating hepatic toxicity associated with FFAs presents a potential therapeutic approach (Wu et al., 2008). The circulation FFA level is usually elevated due to impaired WAT or liver because dysfunctional adipose tissue mass releases more FFA, which is usually overlapped with a decreased FFA clearance. As was noted, an increased level of total FFA in iWAT and serum was observed in the HFF group which indicated an imbalance of FFA release rate and clearance rate, while MHP supplementation improved this condition (Figures 3b–d and 5b) and suppressed the mRNA expression of adipose tissue lipolysis pathway (Figure 3h). In addition, the integrated transcriptomic analysis of liver and adipose tissue revealed that HFF diet significantly inhibited the expression of fatty acid β‐oxidation genes such as *Cpt1a* and *Cpt2*. After MHP treatment, the mRNA levels of these genes returned to normal. Moreover, key genes involved in de novo fatty acid synthesis, such as *Acly* and *Fasn*, were significantly suppressed under the HFF diet (Figure 4g,i), which further explained how the liver–adipose axis worked together to orchestrate the metabolic adaptation.

To maintain metabolic homeostasis, the adipokines and growth factors emanating from the liver–adipose tissue axis have contributed positively (Deng & Scherer, 2010; Fisher & Maratos‐Flier, 2016). WAT plays a crucial role in the development of all aspects of NAFLD by releasing fatty acids, proinflammatory cytokines, and adipokines (Azzu et al., 2020; Du Plessis et al., 2015). Among them, leptin and adiponectin are two extensively investigated ones that are intricately linked with the development and progression of NAFLD (Imajo et al., 2012; Marra & Bertolani, 2009). Adiponectin, which has anti‐inflammatory effect (Polyzos et al., 2011), represents another promising target for the treatment of NAFLD. Resistin functions as a proinflammatory cytokine by promoting the expression of other proinflammatory factors, such as TNF‐α, IL‐6, IL‐1β, and IL‐12 in macrophages and mononuclear cells. Additionally, it can be induced by TNF‐α, IL‐6, and IL‐1 (Bokarewa et al., 2005; Silswal et al., 2005). Chemerin, serving as both an inflammatory chemokine and adipokine, has demonstrated multifaceted involvement in the pathogenesis of metabolic syndrome. Its regulatory roles encompass metabolic inflammation, adipocyte plasticity, and glucose metabolism (Helfer & Wu, 2018). In this study, MHP was shown to restore the mRNA levels of these proinflammatory adipokines in HFF‐fed rats (Figure 5b). Additionally, GSEA analysis reveals that the inflammatory‐related pathways were significantly upregulated in the HFF group, but their expressions were significantly downregulated after MHP treatment, which indicated that MHP may ameliorate inflammation by regulating adipokine secretion. Liu et al. (2021) confirmed the physical interaction between Sparcl1 and TLR4 through immunoprecipitation and demonstrated that Sparcl1 can activate NF‐KB and upregulate the expression of CCL2. Consistently, the serum level of MCP‐1 was significantly elevated in the HFF group but returned to normal levels after MHP treatment (Figure 5c). Therefore, inhibition of SPARCL1 could potentially alleviate chronic metabolic inflammation, and then attenuate hyperlipidemia and hepatic steatosis.

## 5 CONCLUSIONS

In this study, the protective efficacy of the mulberry and *Hippophae*‐based solid beverage MHP against dyslipidemia and hepatic steatosis was confirmed using a high‐fat high‐fructose‐induced rat model of NAFLD. MHP was also shown to inhibit the lipolysis of adipose tissue, reduce the ectopic deposition of lipids in liver tissue, upregulate ACSL1–CPT1a–CPT2 pathway, and promote fatty acid β‐oxidation as revealed by transcriptomics analysis. Additionally, MHP was found to ameliorate metabolic inflammation through regulating the secretion of adipokines. Taken together, our findings provide evidence to support that MHP can improve dyslipidemia and hepatic steatosis and insight into the elucidation of its mechanisms of action.

## AUTHOR CONTRIBUTIONS

**An‐Qi Zhu:** Data curation (equal); formal analysis (equal); investigation (equal); writing – original draft (supporting). **Nin Luo:** Data curation (equal); formal analysis (equal); investigation (equal). **Ling‐Yue Sun:** Data curation (equal); formal analysis (equal); investigation (equal). **Xiao‐Ting Zhou:** Data curation (equal); formal analysis (equal); investigation (equal). **Shi‐Sheng Chen:** Data curation (equal); investigation (supporting). **Zebo Huang:** Conceptualization (equal); project administration (equal); supervision (equal); writing – review and editing (equal). **Xin‐Liang Mao:** Conceptualization (equal); funding acquisition (equal); resources (equal). **Kun‐Ping Li:** Conceptualization (equal); funding acquisition (equal); project administration (equal); supervision (equal); writing – original draft (equal).

## CONFLICT OF INTEREST STATEMENT

Authors X‐ML and S‐SC were employed by the company Perfect Guangdong Co., Ltd. The remaining authors declare that the research was conducted in the absence of any commercial or financial relationships that could be construed as a potential conflict of interest.

## Supporting information


Appendix S1.

## Data Availability

The data that support the findings of this study are available from the corresponding author upon reasonable request.
