# Supplementary material for: Mulberry and Hippophae‐based solid beverage attenuate hyperlipidemia and hepatic steatosis via adipose tissue–liver axis
Source: Food Sci Nutr. 2024 Apr 8;12(7):5052–64. doi: 10.1002/fsn3.4155 (PMC11266884; doi:10.1002/fsn3.4155)
Supplement: Supplementary file 1 — Appendix S1. [file FSN3-12-5052-s001.docx]

**Supplementary materials**

**Table S1.** Test report on nutritional ingredients, heavy metals and microorganisms of MHP ^*^

| **Item** | **Test result** | **Method** |
| --- | --- | --- |
| 1-Deoxynojirimycin (DNJ), mg/100g | 407.87 | GB/T 40642-2021 |
| Moisture, % | 4.78 | GB 5009.3-2016 |
| Ash, % | 5.7 | GB 5009.4-2016 |
| Lead, mg/kg | 0.052 | GB 5009.12-2017 |
| Arsenic, mg/kg | 0.27 | GB 5009.11-2014 |
| Mercury, mg/kg | <0.01 | GB 5009.17-2021 |
| Aerobic bacterial count, CFU/g | <10 | GB 4789.2-2016 |
| Mycete, CFU/g | <10 | GB 4789.15-2016 |
| *Coliform bacteria*, CFU/g | <10 | GB 4789.3-2016 |
| *Staphylococcus aureus*, CFU/25g | Not detected | GB 4789.10-2016 |
| Salmonella, 0/25g | Not detected | GB 4789.4-2016 |
| Energy, kJ/100g | 1237 | GB 28050-2011 |
| Carbohydrate, g/100g | 50.46 | GB 28050-2011 |
| Dietary fiber, g/100g | 31.6 | GB/T 22224-2008 |
| Fat, g/100g | 1.5 | GB 5009.6-2016 |
| Protein, g/100g | 5.96 | GB 5009.5-2016 |

*MHP was produced in a pilot plant that adhered to the strict guidelines of Good Manufacturing Practice. The nutritional ingredients, heavy metals and microorganisms were tested following a series of national standards of China for food safety and quality control as indicated.


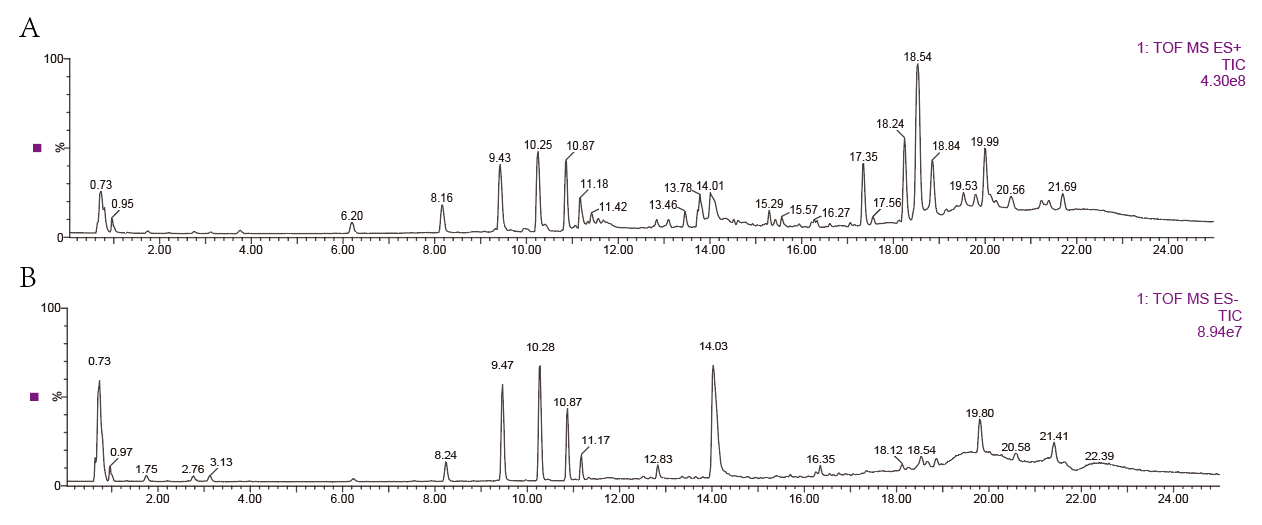


**Figure S1**. Representative LC-ESI-Q-ToF-MS total ion chromatogram of MHP. ESI(+)-MS (A) and ESI(-)-MS (B).

**Table S2.** The main components of MHP identified by LC-ESI(+)/ESI(-)-MS^*^

| No. | Retention Time | Observed m/z | Molecule Formula | Adduction | Compound |
| --- | --- | --- | --- | --- | --- |
| 1 | 1.76 | 163.0123 | C_6_H_13_NO_4_ | [M-e]^+^ | DNJ or Isomer |
| 2 | 2.74 | 163.0123 | C_6_H_13_NO_4_ | [M-e]^+^ | DNJ or Isomer |
| 3 | 3.13 | 163.0123 | C_6_H_13_NO_4_ | [M-e]^+^ | DNJ or Isomer |
| 4 | 6.20 | 453.3181 | Unknown | [M+H]^+^ | Unknown |
| 5 | 8.16 | 566.4062 | / | [M+H]^+^ | Unknown |
|  | 8.24 | 610.42 |  | [M+HCOO]^-^ |  |
| 6 | 9.43 | 679.4976 | / | [M+H]^+^ | Unknown |
|  | 9.47 | 723.4863 |  | [M+HCOO]^-^ |  |
| 7 | 10.25 | 792.5834 | / | [M+H]^+^ | Unknown |
|  | 10.28 | 836.5433 |  | [M+HCOO]^-^ |  |
| 8 | 10.87 | 905.6743 | / | [M+H]^+^ | Unknown |
|  | 10.87 | 949.6023 | / | [M+HCOO]^-^ |  |
| 9 | 11.17 | 1062.6633 | / | [M+HCOO]^-^ | Unknown |
|  | 11.18 | 1018.7567 |  | [M+H]^+^ |  |
| 10 | 12.83 | 401.1253 | C_25_H_22_O_5_ | [M-H]^-^ | Inophyllum E |
| 11 | 13.78 | 318.2751 | C_16_H_12_O_6_ | [M+NH_4_]^+^ | Tectorigenin or Rhamnocitrin |
| 12 | 14.01 | 411.1793 | C_30_H_50_ | [M+H]^+^ | Squalene |
| 13 | 15.57 | 594.14 | C_38_H_56_O_4_ | [M+NH_4_]^+^ | Campesteryl ferulate or Sitogluside |
| 14 | 17.35 | 263.2095 | C_15_H_12_O_3_ | [M+Na]^+^ | Moscatin |
| 15 | 17.35 | 147.0899 | C_6_H_13_NO_3_ | [M-e]^+^ | Fagomine |
| 16 | 18.54 | 283.257 | / | [M+H]^+^ | Unknown |
| 17 | 18.84 | 313.2476 | C_20_H_40_O_2_ | [M+H]^+^ | Arachic acid |
| 18 | 19.53 | 474.2861 | C_20_H_23_N_7_O_7_ | [M+H]^+^ | Folinic acid |
| 19 | 19.8 | 395.193 | C_28_H_58_ | [M+H]^+^ | Octacosane |
| 20 | 19.99 | 284.2675 | C_19_H_38_ | [M+NH_4_^]+^ | 1-Nonadecene |
| 21 | 20.56 | 341.2737 | C_20_H_20_O_5_ | [M+H]^+^ | Morachalcone A |
| 22 | 21.41 | 283.3141 | C_18_H_36_O_2_ | [M-H]^-^ | Stearic acid or Ethylpalmitate |
| 23 | 21.69 | 312.2989 | / | [M+H]^+^ | Unknown |

^*^ LC-ESI-Q-ToF-MS analysis was performed on Waters Xevo G2 Q-ToF-Mass System using a Waters Xbridge BEH C18 (2.1×100 mm, 2.5μm) column. The column temperature was maintained at 30 ℃. The flow rate and injection volume were set at 0.3 mL/min and 1 μL, respectively. Water containing 0.1% formic acid (A) and acetonitrile (B) were used as mobile phase. For LC-ESI(+)-MS analysis, the capillary voltage and cone voltage were set at 3.0 kV and 40 V, respectively. The ion source temperature and desolvation temperature were maintained at 120 °C and 48 °C, while the cone gas flow and desolvation gas flow were adjusted to 50 L/h and 600 L/h, respectively. For LC-ESI(-)-MS analysis, the capillary voltage and cone voltage were set at 2.5 kV and 40 V, respectively. The ion source temperature and desolvation temperature were maintained at 100 °C and 48 °C, while the cone gas flow and desolvation gas flow were adjusted to 50 L/h and 600 L/h, respectively.


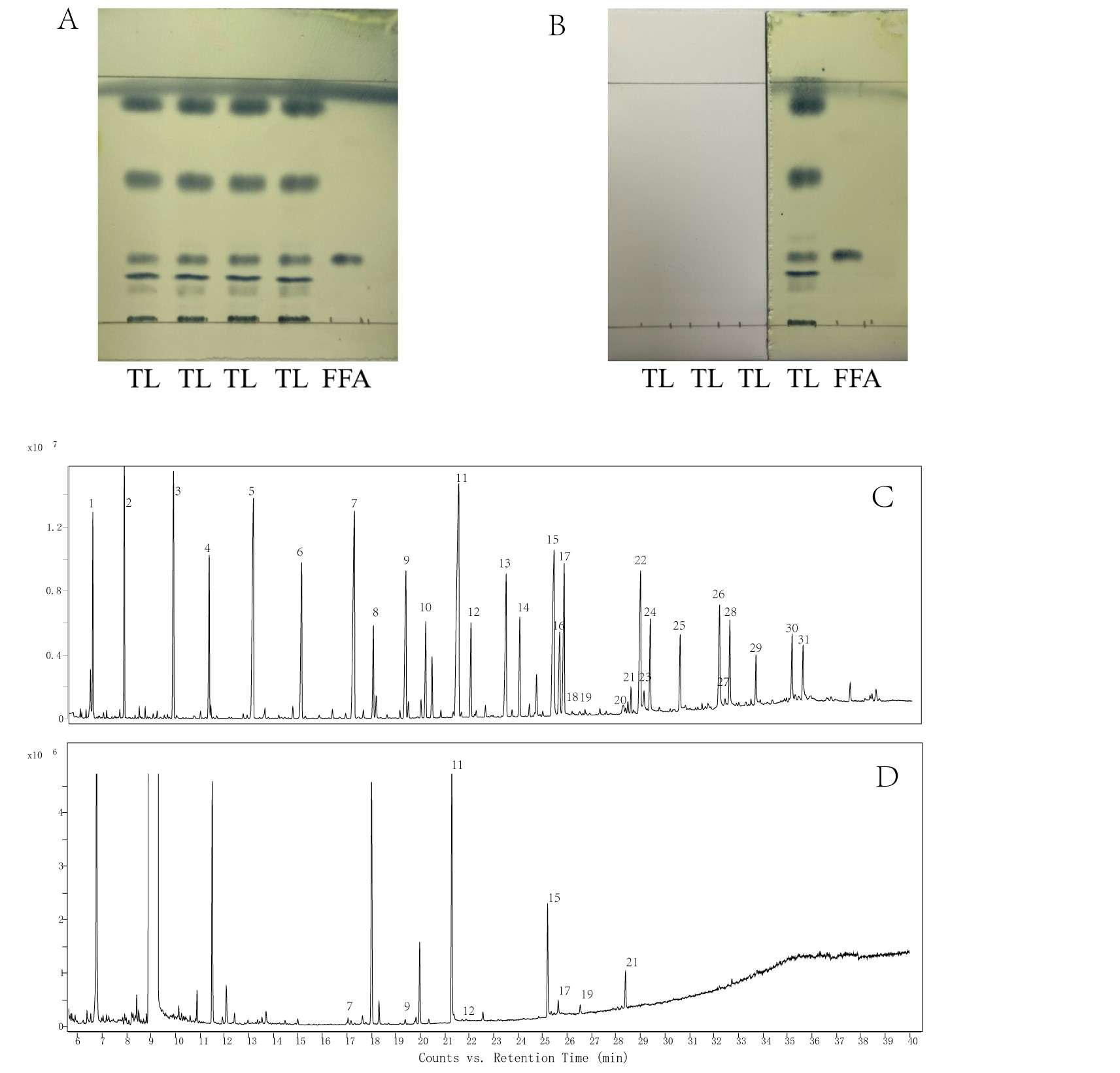


1.C6:0 2.C8:0 3.C10:0 4.C11:0 5.12:0 6.13:0 7.C14:0 8.C14:1 9.C15:0 10.C15:1 11.C16:0 12.C16:1 13.C17:0 14.C17:1 15.C18:0 16.C18:1n9t 17.C18:1n9c 18.C18:2n6t 19.C18:2n6c 20.C18:3n6 21.C18:3n3 22.C20:0 23.C20:1 24.C20:2 25.C21:0 26.C22:0 27.C20:5n3 28.C22:1n9 29.C23:0 30.C24:0 31.C24:1

**Figure S2.** Quantitative profiling of free fatty acids in serum and adipose tissue. For the serum sample, 350 µL of CHCl_3_-MeOH (2:1, v/v) solution was added to 50 µL of serum, vortexed for 30 s, then 300 µL of distilled water was added and vortexed again for 30 s, and finally iced for 5 min. The mixture was centrifuged at 14000 rpm for 5 min at room temperature and 160 µL of the lower organic layer was collected as the total lipid fraction. For the fat tissue sample, 700 µL of CHCl_3_-MeOH (2:1, v/v) solution was added to 25 mg of adipose tissue, and homogenized for 4 min, then 300 μL of distilled water was added and vortexed for 30 s and centrifuged at 14000 rpm for 5 min, and 160 µL of the organic layer was collected as the total lipid portion. FFA was fractionated by developed on HPTLC silica gel plates and linoleic acid reference was spotted as mark (A). After developing with a solvent mixture of n-hexane: diethyl ether: acetic acid (70:30:1, v/v/v), a side by side confirming of FFA by chromogenic reaction was performed using linoleic acid reference. Then, the FFA band was scraped from the uninvolved silica gel plate and resuspended with CHCl_3_-MeOH (2:1, v/v) solution. After centrifugation, the 50 µL of the supernatant was pipetted and derivatized with 40 µL of 3-(trifluoromethyl) phenyltrimethyl ammonium hydroxide at room temperature for 1 h. for GC-MS analysis (B). Quantitative profiling of FFA was performed on 7890B-5977B GC-MS System with a DB-23 column (60 m × 0.25 mm × 0.15 µm) (Agilent, MA, USA). The temperature programming was set to 50 ℃, 0.5 min → 50–140 ℃ (20 ℃/min), 5 min → 140–240 ℃ (4 ℃/min), 5 min. The auxiliary temperature was 240 ℃ and the injector temperature was 250 ℃ with 1.0 µL injection volume (10:1 split ratio). The helium carrier gas flow rate was set at 1 mL/min. MS detection was used full ion scanning m/z 40–500 amu. The EI source temperature was 230 ℃. The MS quadrupole temperature was maintained at 150 ℃. A commercial fatty acid methyl esters mixture, F.A.M.E. Mix (C4-C24, Supelco, USA), was used as standard (C). A representative total ion chromatograph of FFA was shown in (D). MassHunter Qualitative Analysis B.07.00 and Quantitative Analysis B.07.00 were used for quantitative analysis.

**Table S3.** The composition of D12492*

| **Product #D12492** | **gm%** | **kcal%** |
| --- | --- | --- |
| Protein | 26.2 | 20 |
| Carbohydrate | 26.3 | 20 |
| Fat | 34.9 | 60 |
| Total kcal/gm | 5.24 | |
| Ingredient | gm | kcal |
| Casein, 30 Mesh | 200 | 800 |
| L-Cystine | 3 | 12 |
| Corn Starch | 0 | 0 |
| Maltodextrin 10 | 125 | 500 |
| Sucrose | 68.8 | 275.2 |
| Cellulose, BW200 | 50 | 0 |
| Soybean Oil | 25 | 225 |
| Lard | 245 | 2205 |
| Mineral Mix S10026 | 10 | 0 |
| Dicalcium Phosphate | 13 | 0 |
| Calcium Carbonate | 5.5 | 0 |
| Potassium Citrate, 1 H2O | 16.5 | 0 |
| Vitamin Mix V10001 | 10 | 40 |
| Choline Bitartrate | 2 | 0 |
| FD&C Blue Dye #1 | 0.05 | 0 |
| Total | 773.85 | 4057 |

* The data is presented by Research Diets Inc., New Brunswick, NJ, USA.

**Table S4.** The composition of normal chow diet

| **Vitamin** | **Mineral** | | **Amino acid** | | |
| --- | --- | --- | --- | --- | --- |
| Vitamin A (7800.00 IU) | Sodium (3.10 g) | | Methionine+Cystine (5.80 mg) | | |
| Vitamin D (1200.00 IU) | Magnesium (2.90 g) | | Lysine (8.90 g) | | |
| Vitamin E (67.00 mg) | Kalium (7.40 g) | | tryptophan (2.10 g) | | |
| Vitamin K (5.00 mg) | Cuprum (11.40 mg) | | Arginine (9.90 g) | | |
| Vitamin B1 (10.00 mg) | Iron (113.70 mg) | | Leucine (14.80 g) | | |
| Vitamin B2 (15.00 mg) | manganese (80.00 mg) | | Isoleucine (7.40 g) | | |
| Vitamin B6 (10.00 mg) | Zinc (31.60 mg) | | Threonine (6.60 g) | | |
| Vitamin B12 (0.02 mg) | Selenium (0.20 mg) | | Valine (8.90 g) | | |
| Niacin (55.00 mg) | Iodine (0.70 mg) | | Histidine (4.90 g) | | |
| Pantothenic acid (22.00 mg) |  | | Phenylalanine+Tyrosine (14.60 g) | | |
| Biotin (0.20 mg) |  | |  | | |
| Choline (1250.00 mg) |  | |  | | |
| Folate (6.60 mg) |  | |  | | |
| Energy supply ratio | Protein | Fat | | Carbohydrate | Total |
|  | 23.07% | 11.85% | | 65.08% | 3.40kcal/g |

* The data is presented by Beijing Keao Xieli Feed Co., Ltd, Beijing, China.


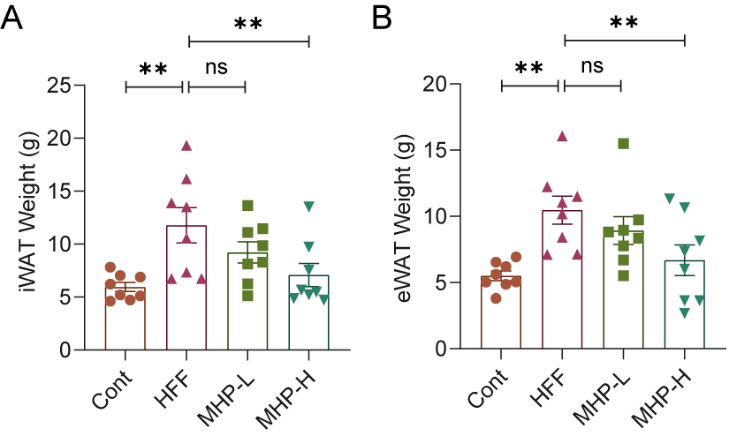


**Figure S3**. MHP reduces the fat mass in HFF-fed rats. (A-B) Measurement of mass of inguinal white adipose tissue (iWAT) and epididymal white adipose tissue (eWAT). Data are expressed as mean ± SEM (n = 8). **p* < 0.05, ***p* < 0.01vs. HFF group.


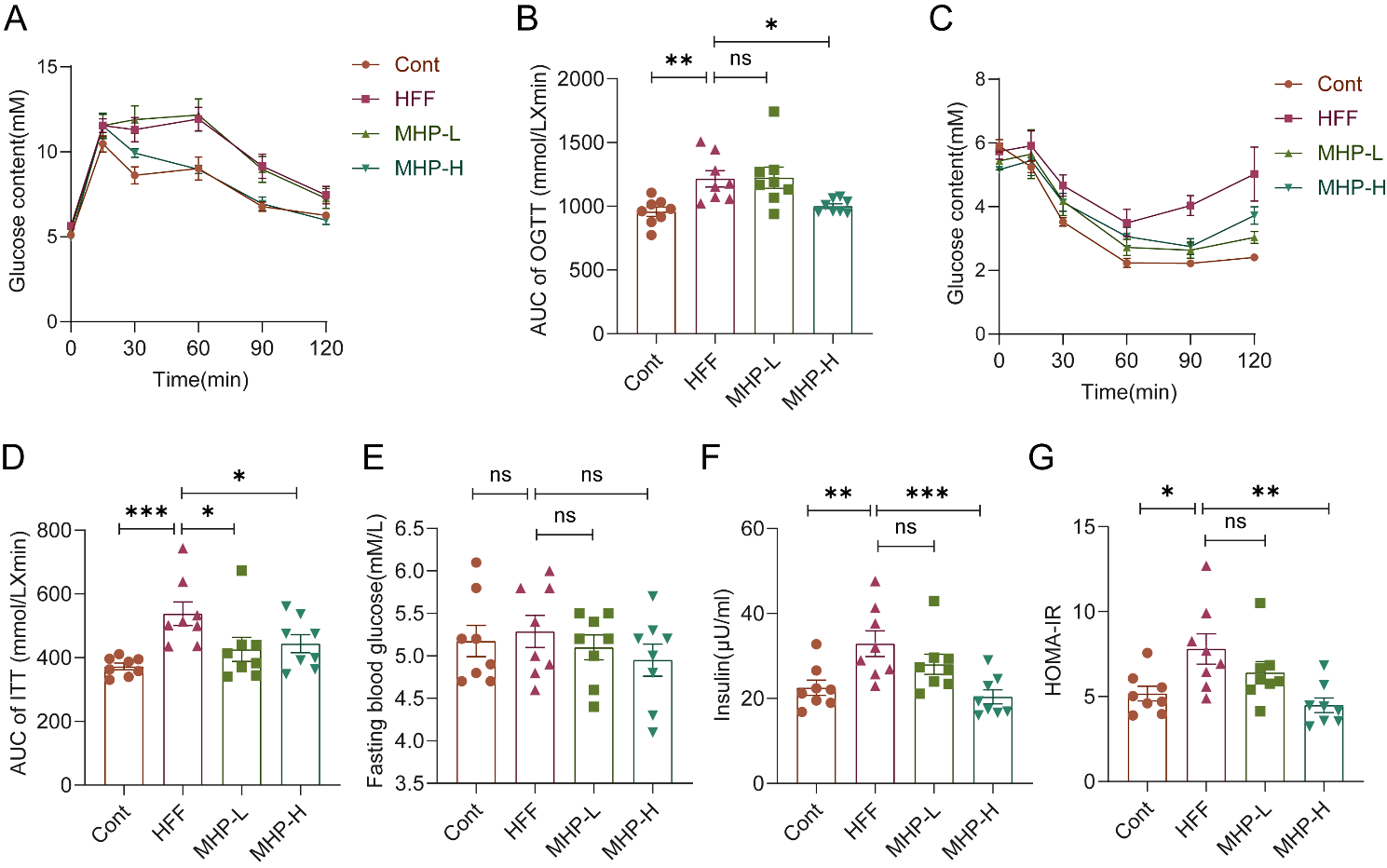


**Figure S4**. MHP ameliorates insulin resistance induced by high-fat high-fructose diet. (A&B) Oral glucose tolerance test (OGTT) of experimental rats and glucose area under the curve (AUC). (C&D) Insulin tolerance test (ITT) and glucose AUC during ITT. (E-F) Fasting blood glucose (FBG) level, serum insulin level and homeostasis model assessment of insulin resistance (HOMA-IR) index of the experimental rats. Data are expressed as mean ± SEM (n = 8). **p* < 0.05, ***p* < 0.01, ****p* < 0.001 vs. HFF group.
